# Editorial: Traditional Sporting Games and Play: Enhancing Cultural Diversity, Emotional Well-Being, Interpersonal Relationships and Intelligent Decisions

**DOI:** 10.3389/fpsyg.2021.766625

**Published:** 2021-10-29

**Authors:** Pere Lavega-Burgués, Marco Antonio Coelho Bortoleto, Miguel Pic

**Affiliations:** ^1^National Institute of Physical Education of Catalonia, University of Lleida, Lleida, Spain; ^2^Physical Education and Humanities (DEFH), University of Campinas, Campinas, Brazil; ^3^Institute of Sport, Tourism, and Service, South Ural State University Chelyabinsk, Chelyabinsk, Russia

**Keywords:** traditional sporting games, culture, emotions, decision learning, motor praxeology

## Summary

**General Approach. Motor Praxeology, A New Discipline for Researching Traditional Sporting Games**

- Book Review: Contribution à un Lexique Commenté en Science de l'Action Motrice. Zhaïra Ben Chaâbane.
- Book Review: Éléments de Sociologie du Sport. Bordes Pascal.
- Book Review: La Aventura Praxiológica. Ciencia, Acción y Educación Física. María Pilar Founaud and Asier Oiarbide.
- The Universals of Games and Sports. Pierre Parlebas.
- Book Review: Games and Society in Europa. Bartosz Prabucki.
- Book Review: Els jocs i els esports Tradicionals. Tradicionari. [The Traditional Games and Sports. Traditionari]. Gabriel Pubill.
- Book Review: Recreios Collegiaes. Mário Duarte Maia Rodrigues.

**Traditional Sporting Games and Play: Culture, and Diversity:**

- Traditional Games as Cultural Heritage: The Case of Canary Islands (Spain) From an Ethnomotor Perspective. Rafael Luchoro-Parrilla, Pere Lavega-Burgués, Sabrine Damian-Silva, Queralt Prat, Unai Sáez de Ocáriz, Enric Ormo-Ribes and Miguel Pic.
- Playing Ludomotor Activities in Lleida During the Spanish Civil War: An Ethnomotor Approach. Enric Ormo-Ribes, Pere Lavega-Burgués, Rosa Rodríguez-Arregi, Rafael Luchoro-Parrilla, Aaron Rillo-Albert and Miguel Pic.
- The Commemoration of Independence Day: Recalling Indonesian Traditional Games. Mustika Fitri, Hana Astria Nur and Wulandari Putri.
- The Game of Skittles on the Northern Route of the Camino de Santiago.José E. Rodríguez-Fernández, Mar Lorenzo-Moledo, Jesús García-Álvarez and Gabriela Míguez-Salina.
- Festival Traditional Sports and Games: Intercultural Dialog, Sustainability, and Empowerment. Soraia Chung Saura and Ana Cristina Zimmermann.
- The Blows and Capoeira Movements from the Caricatures of Calixto Cordeiro. Paulo Coêlho Araújo and Ana Rosa Jaqueira.
- Book Review: The Story of Catch: The Story of Lancashire Catch-as-Catch-Can Wrestling. Kazimierz Waluch.

**Traditional Sporting Games and Play and Emotional Well-Being. A Sociocultural Approach**

- Traditional Sporting Games as Emotional Communities: The Case of Alcover and Moll's Catalan–Valencian–Balearic Dictionary. Antoni Costes, Jaume March-Llanes, Verónica Muñoz-Arroyave, Sabrine Damian-Silva, Rafael Luchoro-Parrilla, Cristòfol Salas-Santandreu, Miguel Pic and Pere Lavega-Burgués.
- The Emotional States Elicited in a Human Tower Performance: Case Study. Sabrine Damian-Silva, Carles Feixa, Queralt Prat, Rafael Luchoro-Parrilla, Miguel Pic, Aaron Rillo-Albert, Unai Sáez de Ocáriz, Antoni Costes and Pere Lavega-Burgués.
- Emotional Well-being and Traditional Cypriot Easter Games: a mixed method analysis. Christiana Koundourou, Markella Ioannou, Chara Stephanou, Maria Paparistodemou and Theodora Katsigari.

**Traditional Sporting Games and Play and Emotional Well-Being. A Pedagogical Approach**

- The Effect of Traditional Opposition Games on University Students' Mood States: The Score and Group Type as Key Aspects. María Isabel Cifo Izquierdo, Verónica Alcaraz-Muñoz, Gemma Maria Gea-García, Juan Luis Yuste-Lucas and José Ignacio Alonso Roque.
- Student Moods Before and After Body Expression and Dance Assessments. Gender Perspective. Mercè Mateu, Silvia Garcías, Luciana Spadafora, Ana Andrés and Eulàlia Febrer.
- Joy in Movement: Traditional Sporting Games and Emotional Experience in Elementary Physical Education. Verónica Alcaraz-Muñoz, María Isabel Cifo Izquierdo, Gemma Maria Gea García, José Ignacio Alonso Roque and Juan Luis Yuste Lucas.
- New Images for Old Symbols: Meanings That Children Give to a Traditional Game. Alfonso García-Monge, Henar Rodríguez-Navarro and Daniel Bores-García.

**Traditional Sporting Games and Decision Learning**

- Sports Teaching, Traditional Games, and Understanding in Physical Education: A Tale of Two Stories. Raúl Martínez-Santos, María Pilar Founaud, Astrid Aracama and Asier Oiarbide.
- Book Review: La paradoja de jugar en tríada. El juego motor en tríada.Raúl Martínez-Santos.
- Influence of Traditional Sporting Games on the Development of Creative Skills in Team Sports. The Case of Football. Alexandre Oboeuf, Sylvain Hanneton, Joséphine Buffet, Corinne Fantoni and Lazhar Labiadh.

The editors of this e-book are pleased to introduce the Research Topic “Traditional Sporting Games and Play: Enhancing Cultural Diversity, Emotional Well-being, Interpersonal Relationships and Intelligent Decisions.”

This is the first time that different research groups and researchers from different countries (Argentina, Brazil, France, Indonesia, Poland, Portugal, Russia, and Spain) and continents (America, Asia, and Europe) have shared a scientific challenge: to show through scientific evidence that traditional sporting games (TSGs) are worthy objects of study. The 24 texts that form part of this work show the polyhedral nature of TSGs from different domains of knowledge. Shared relationships, cultural meanings, emotions, and intelligent learning are different dimensions of the same phenomenon: the traditional sporting game. Frontiers in Psychology recognizes this multimodal nature of TSGs by integrating the articles in this book into the following sections: Movement Science and Sport Psychology, Personality and Social Psychology, Educational Psychology, Cultural Psychology, and Educational Psychology.

This e-book shows that the TSG operates as a mirror of the society where it is hosted, while at the same time it functions as an authentic laboratory of life experiences that have an impact on the deepest part of its players. Far from being minor manifestations, the texts confirm their worthiness and function in different past and present societies. Applied research is also presented in the context of formal education (primary, secondary, and university physical education), as well as in informal education (festive contexts) as well as in the field of sports initiation.

At the same time, this book also presents a wide variety of disciplines in researching TSGs: anthropology, ethnology, ethnography, sociology, history, pedagogy, social psychology, and a not very well-known discipline in the English-speaking scientific field: motor praxeology, the science of motor action.

At the same time, the scientific research on TSGs also shows a great variety of methodological strategies, from quasi-experimental designs to case studies or ethnographies. The research groups involved in this book have used quantitative approaches, qualitative studies, and as mixed-method designs. Statistical analyses (using different techniques of inferential statistics) and also content analyses of narrative texts (with different specific programs) reveal the distinctive features of TSGs and their contribution to a systemic view of health, i.e., physical, emotional, relational, and cognitive well-being.

Motor praxeology was created more than 50 years ago by Pierre Parlebas, a former student and professor of the University of Paris (Sorbonne—France). His unique academic journey includes higher education in physical education and sports, sociology, psychology, mathematics, and linguistics. Parlebas could be considered the Darwin, Freud, or Einstein of the field of physical education and sports, because he created a specific epistemological corpus for the field of motor practices in general and TSGs in particular. The theory of motor action bases its scientific foundations on a structural rather than structuralist approach that is original to the field of physical activity and sport.

Thanks to this publication in e-book format, researchers in the English-speaking world will be able to find out a new approach to researching TSGs, based on the systemic or structural approach. We hope that our colleagues in the Anglosphere will join us in the knowledge and application of the fundamentals of this scientific discipline, as has already been done in Africa (e.g., Algeria, Congo, Guinea, Mali, Morocco, and Tunisia), America (e.g., Argentina, Brazil, Colombia, Chile, and United States), Asia (e.g., South Korea), and Europe (e.g., France, Italy, Portugal, Spain, and Switzerland).

For this reason, it is not surprising that some authors of this book review the most important of Parlebas' books. Chaâbane takes on the challenge of reviewing of probably the main publication in the science of motor action: *Contribution à un lexique commenté en science de l'action motrice*. Through the definition of the core concepts for an in-depth understanding of motor situations, Parlebas proposes a specific scientific language to the domain of physical activities and sports (PAS) and an innovative analysis of physical and playful activities. The first edition (1981) is a testimony of the birth of a new point of view on motor actions. The second one (1999) highlights one of the consequences of this new approach: the interest in traditional games. Parlebas presents the concept of the internal logic (Parlebas, 1981, 1999), an intrinsic reality of sporting games which exposes the motor conducts. These conducts manifest according to the relationship they create between the actor and its environment: relationship with space, objects, time, and other actors. This discipline also offers a systemic and scientific classification of TSG that has provided multiple interpretations and applications. In addition, the motor action states that, on the basis of a rigorous analysis of ludomotor structures, extrinsic elements of the game bring further clarifications and enrich the understanding of TSG, from the angle of their relationship to culture and the social environment in which they are developed. Every motricity is an “ethnomotricity” (Parlebas, 1981, 1999). The frivolousness of TSGs is only an appearance: they are in reality the mirror of the community to which they belong, and they participate in the cultural identity of each society which represents original playful patterns, linked to their lifestyles (Parlebas, 2001; Lagardera and Lavega, 2003).

Pascal Bordes writes the book review of Parlebas' second key work: Éléments de Sociologie du Sport. Parlebas uses a rigorous methodology linked to an innovative standpoint to develop his scientific contributions. Sport is coherently conceived and understood as “the finite and countable set of motor situations codified in the form of institutionalized competition” (p. 55). Any traditional sporting game is precisely distinguished from sports because it lacks recognition by the authorities in place, and from federations and international committees in particular. They represent the diversity of an exuberant ludomotor heritage. On the contrary, sports embody massive hierarchical, centralized, and normative regulatory systems.

Behind the apparent diversity of forms in the sports world there operates one same deep structure: opposition in the form of contests between teams or individuals that fight on equal terms according to scores of capitalizable numerical units; sport presents itself as the Esperanto of physical games.

Subsequently, Founaud and Oiarbide present the book review of La Aventura Praxiológica. Ciencia, Acción y Educación Física that contains 32 selected and translated by the editor Raúl Martínez-Santos, who groups them chronologically and thematically. It is the adventure that Parlebas undertakes to create the science of motor action, a new conception of physical education, the process of creation of a matrix science, a change of paradigm for an orphan, as physical education has been.

By reading these three core books of motor praxeology, researchers will recognize the fundamentals and use of the main meaningful concepts related to the system and the actor, i.e., the game and its protagonists. Concerning the game, two key concepts emerge: (a) the *internal logic* or identity card, that asks players to solve four types of internal relationships: with others, with the physical space, with time, and with the materials; (b) *an innovative classification* of sporting games with eight different action domains regarding the relationships that agents establish with their social and natural milieus. These two concepts, which are widely used as independent variables in multiple research studies, lead us to the key concept of the actor: *the “*motor conduct” understood as the meaningful organization of motor behaviors, as the common factor of games, sports, and any physical activity, and, consequently, as the proper object of object physical education.

The reader of this e-book can feel privileged accessing the article The Universals of Games and Sports written especially for this monograph by Pierre Parlebas. This article shows that behind the superficial disorder that is all the rage in traditional games, there is an in-depth order in there as well. The “universals” are these laws of order, these underlying objective systems on top of which the praxic exchanges that can be observed in all games and sports are built: operational models which represent the basic structures of the functioning of any sporting game, bearers of the fundamental features of its internal logic. Ludodiversity is a confirmed phenomenon: the analysis of universals reveals that the alleged superior complexity of sports is an illusion. Between traditional games and sports there is not a difference in degree, but in nature.

This e-book also reviews other works that have used motor praxeology to research TSGs: Prabucki, Poland reviews Games and Society in Europe (published by the European Traditional Sports and Games Association (ETSGA/AEJeST) stating the status of TSGs as an intangible cultural heritage.

Pubill analyses the Catalan Encyclopedia The Traditional Games and Sports. Traditionari. The 25 authors provide an excellent approach from the past (tradition) and the present (modernity) in order to understand the current society through the foster important values on TSGs.

Rodrigues describes how motor praxeology also contributes to understanding the motor and socio-cultural richness of Portuguese TSGs, in the work Recreios Collegiaes, authored by Priest Pedro Aloy. One of the findings of the analysis of this work is the great predominance of socio-motor games that encourage a great diversity of interpersonal relationships.

The fundamentals of motor praxeology also allow to identify the ethnomotor singularity of TSGs in the Canary Islands (Luchoro-Parrilla et al.). The playful activities were games (activities with rules) and played with objects, that enhanced material and social sustainability experiences. Motor action theory was also applied to interpret the distinctive features of TSGs during the Spanish Civil War (Ormo-Ribes et al.). Different features were observed when comparing TSGs with and without war connotations.

Fitri et al. present an ethnography of TSGs in the commemoration of Indonesia Independence Day that is conducted annually. Games and local culture are an inseparable binomial.

Rodríguez-Fernández et al. analyze, using mixed methods, the Skittles on the Northern Route of the Camino de Santiago, revealing symbols of local traditional heritage. Saura and Zimmermann wrote an article to discuss how a TSG Festival in a public school in São Paulo, Brazil, promotes intercultural dialog with a focus on sustainability, and how it empowers people and creates equality among its players.

Araújo and Jaqueira apply a multi-method process to analyze the Blows and Capoeira Movements from the Caricatures of Calixto Cordeiro in Brazil. The research shows substantial information about the blows/moves of this contest, as well as its different names, slang, and expressive forms, often associated with different groups of practitioners from different Brazilian cities.

The contextual view of TSGs is complemented by the contribution of Waluch, by reviewing the book The Story of Catch: The Story of Lancashire Catch-as-Catch-Can Wrestling by Ruslan C. Pashayev. The historic approach describes this wrestling TSG in different periods. This is the only major book ever to cover this subject.

This e-book explains that emotions can be studied from different disciplines, in addition to psychology. Costes et al. explain how the TSGs collected in Alcover and Moll's Catalan-Valencian-Balearic Dictionary originated playful communities of emotions among their protagonists. Damian-Silva et al. conduct an ethnographic study to reveal the emotional states elicited in a human tower performance. Emotional well-being also plays a role in the mixed-method study around the traditional Cypriot Easter Games written by Koundourou et al. The findings indicate that the games elicit emotions such as joy, excitement, and euphoria. Emotions such as embarrassment, frustration, and anger are also observed occasionally, specifically in situations of competitiveness and defeat.

TSGs may trigger positive effects on mood states in different families of TSG. Cifo et al. focus on traditional opposition games and observe that competition and the gender of the group are variables to be taken into account. Mateu et al. research the moods of male and female students in their body language and dance choreographies. Alcaraz-Muñoz et al. study the joy in TSGs practiced by elementary physical education students. This study brings the value of considering games as a key role to promote the education of social-emotional well-being in schoolchildren, as the basis of academic training

García-Monge et al. explore the childhood meanings of the chained bear TSG and compare their findings with the cultural meanings of different traditional versions of this game. Their results show that, beyond the individual images that each child created in their mind, most of them coincided in stories about harassment, defense, theft, and protection.

The pedagogical use of TSGs is also the focus of interest in the contribution by Martínez-Santos et al. when they carry out a conceptual analysis to try to and reconcile two perspectives, namely motor praxeology and teaching games for understanding (TGfU). Their conclusion is that TGfU, or game-based approaches to sports coaching and teaching, can take great advantage of the motor-praxeological rationale.

Martínez-Santos reviews the book *La paradoja de jugar en tr*í*ada* (The motor game in triad) by Pic and Navarro-Adelantado, showing the sporting games in which three teams play against each other performing interesting paradoxical situations. Any game in which three agents interact leads to triadic motor interaction systems, that is, to motor action systems in which one player's acts must be interpreted as positive or negative in relation to any other two players' relationship.

Finally, Oboeuf et al. illustrate the influence of traditional sporting games on the development of creative skills in football. Creativity originates from an interaction between divergence and convergence. In this research, the number of communications (fluidity) and the diversity of updated communications (flexibility) are the divergence indicators. Convergence is studied as the ability to make good decisions. The results show that TSGs can help develop players' creative abilities.

The editors would also like to acknowledge the valuable work of around fifty reviewers that joined this process. With this great team, we believe this book represents a substantial contribution to the TSG field.

Finally, we would also like to thank the journal Frontiers in Psychology for being a pioneer in organizing a scientific monograph on traditional sporting games and play.

## Author Contributions

All authors listed (PL-B, MB, and MP) have made a substantial, direct, and intellectual contribution to the work, and approved it for publication.

## Conflict of Interest

The authors declare that the research was conducted in the absence of any commercial or financial relationships that could be construed as a potential conflict of interest.

## Publisher's Note

All claims expressed in this article are solely those of the authors and do not necessarily represent those of their affiliated organizations, or those of the publisher, the editors and the reviewers. Any product that may be evaluated in this article, or claim that may be made by its manufacturer, is not guaranteed or endorsed by the publisher.
